# Supplementary material for: EASY-NET Program: Methods and Preliminary Results of an Audit and Feedback Intervention in the Emergency Care for Acute Myocardial Infarction in the Lazio Region, Italy
Source: Healthcare (Basel). 2023 Jun 5;11(11):1651. doi: 10.3390/healthcare11111651 (PMC10252972; doi:10.3390/healthcare11111651)

# Supplementary Material


Table S1. Type of information collected in the “Audit form”. ........ 2

Table S2. ICD-9-CM codes ........ 3

Table S3. Link to indicators protocols for AMI pathways ........ 5

Table S4. Link to indicators protocols for ischemic stroke pathways ........ 5

Figure S1 and Table S5: Number of hospitalizations of patients with STEMI by facility (2021). ........ **Error! Bookmark not defined.**

Figure S2 and Table S6: Proportion of patients with STEMI treated with PTCA within 90 min from access to ER of total treated with PTCA within 12 h, by facility (2021). ........ **Error! Bookmark not defined.**

Figure S3 and Table S7: Mortality within 30 days after first admission to hospital for AMI, by facility (2021). ........ 6

Figure S4 and Table S8: In-hospital mortality within 30 days after first admission to hospital for STEMI, by facility (2021). ........ **Error! Bookmark not defined.**

Figure S5 and Table S9: In-hospital mortality within 30 days after first admission to hospital for AMI, by facility (2021). ........ 7

Figure S6: Proportion of patients with STEMI treated with PTCA within 90 min from access to ER, by facility (2021). A: facility in volume class 0-10, B: facility in volume class 11-50, C: facility in volume class 51-100, D: facility in volume class 101-150, E: facility in volume class >=150. ........ 8

Figure S7: Mortality within 30 days after first admission to hospital for STEMI, by facility (2021). A: facility in volume class 0-10, B: facility in volume class 11-50, C: facility in volume class 51-100, D: facility in volume class 101-150, E: facility in volume class >=150. ........ 9

| **Requested Information** | **Responses** |
|---|---|
| **Was an Audit conducted on the results of the last monitoring?** | Yes<br>No |
| **Which of the following actions were implemented in preparing and conducting the Audit?** | Identification of all stakeholders to be involved<br>Convening the Audit meeting<br>Discussion of the results of the monitoring indicators<br>Presentation and discussion of clinical cases<br>Identification of improvement actions<br>Identification of those responsible for the implementation of improvement actions<br>Clear identification of time for verification of implemented actions |
| **Has an Audit Manager been identified?**<br><br>(If yes, please indicate the Audit Manager profession/discipline and role in the Structure) | Yes<br>No |
| **Has a Multidisciplinary Audit Team been established?**<br><br>(If yes, please indicate the composition of the Multidisciplinary Team) | Yes<br>No |
| **Were additional indicators calculated and discussed compared to those contained in the last report submitted?**<br><br>(If yes, please indicate the additional indicators considered in the Audit, the reference year for the calculation and the result.) | Yes<br>No |
| **Overall, were quality indicators identified that deviate from the standard value and/or the regional reference value?**<br><br>(If yes, please report the indicators that deviate from the reference value) | Yes<br>No |
| **The overall results of the evaluation were disseminated to the healthcare professionals involved in the care pathway in question?**<br><br>(If yes, indicate the professionals to whom the reports were sent e.g. doctors, nurses, etc.) | Yes<br>No |
| **Was an audit report produced?**<br><br>(If yes, please indicate the professionals to whom the report was sent) | Yes<br>No |
| **What improvement actions have been identified?**<br>**For each indicator showing deviations from the reference values, identify one or more improvement actions.**<br><br>Specify the timeframe in which the level of implementation of the identified improvement actions will be verified? (e.g. 1 - 2 months; 3 - 4 months; 5 - 6 months) | Free response |
| **Depending on the presence and degree of the identified deviation, were identified structure objectives to be achieved for the indicators considered?** | Yes<br>No |

| | |
|---|---|
| (If yes, please report these objectives in relation to the indicators) | |

**Table S1.** Type of information collected in the "Audit form".

**Table S2.** ICD-9-CM codes.

| Indicators | Calculation | Data sources | ICD9-CM primary or secondary diagnoses codes | | ICD9-CM procedures codes |
|---|---|---|---|---|---|
| | | | Inclusion | Exclusion | Inclusion |
| AMI: volume of hospitalizations | Number of hospitalizations of patients diagnosed with AMI | HDR | 410.9X | | |
| STEMI: volume of hospitalizations | Number of hospitalizations of patients diagnosed with STEMI | HDR | 410.XX | 410.7X, 410.9.X | |
| STEMI: volume of hospitalizations with at least one PTCA | Number of hospitalizations of patients diagnosed with STEMI who received at least one PTCA | HDR | 410.XX | 410.7X, 410.9.X | 00.66, 36.01, 36.02, 36.05, 36.06, 36.07 |
| STEMI: proportion treated with PTCA within 90 minutes | Number of hospitalized patients diagnosed with STEMI who received PTCA within 90 minutes from access to ER / Number of hospitalization of patients diagnosed with STEMI | HDR, HEIS | 410.XX<br><br>411, 413, 414, 423.0, 426, 427, 428, 429.5, 429.6, 429.71, 429.79, 429.81, 518.4, 518.81, 780.01, 780.2, 785.51, 799.1, 997.02, 998.2 | 410.7X, 410.9.X 427.5 | 00.66, 36.01, 36.02, 36.05, 36.06 36.07 |
| STEMI: proportion of PTCA within 90 minutes of those treated with PTCA within 12h | Number of hospitalized patients diagnosed with STEMI who received PTCA within 90 minutes from access to ER / Number of hospitalization of patients diagnosed with STEMI who received PTCA within 12 hours | HDR, HEIS | 410.XX<br><br>411, 413, 414, 423.0, 426, 427, 428, 429.5, 429.6, 429.71, 429.79, 429.81, 518.4, 518.81, 780.01, 780.2, 785.51, 799.1, 997.02, 998.2 | 410.7X, 410.9.X 427.5 | 00.66, 36.01, 36.02, 36.05, 36.06 36.07 |

| | | | | | |
|---|---|---|---|---|---|
| AMI: in-hospital mortality | Number of hospitalized patients diagnosed with AMI who died during the hospital stay / Number of hospitalized patients diagnosed with AMI | HDR, HEIS, Tax Registry | 410.9X | | |
| STEMI: in-hospital mortality | Number of hospitalized patients diagnosed with STEMI who died during the hospital stay / Number of hospitalized patients diagnosed with STEMI | HDR, HEIS, Tax Registry | 410.XX<br><br>411, 413, 414, 423.0, 426, 427, 428, 429.5, 429.6, 429.71, 429.79, 429.81, 518.4, 518.81, 780.01, 780.2, 785.51, 799.1, 997.02, 998.2 | 410.7X, 410.9.X 427.5 | 00.66, 36.01, 36.02, 36.05, 36.06 36.07 |
| AMI: 30-day mortality | Number of hospitalized patients diagnosed with STEMI who died during the 30 days after hospital discharge/ Number of hospitalized patients diagnosed with AMI | HDR, HEIS, Tax Registry | | | |
| STEMI: 30-day mortality | Number hospitalized patients diagnosed with STEMI who died during the 30 days after hospital discharge / Number of hospitalized patients diagnosed with STEMI | HDR, HEIS, Tax Registry | 410.XX<br><br>411, 413, 414, 423.0, 426, 427, 428, 429.5, 429.6, 429.71, 429.79, 429.81, 518.4, 518.81, 780.01, 780.2, 785.51, 799.1, 997.02, 998.2 | 410.7X, 410.9.X 427.5 | 00.66, 36.01, 36.02, 36.05, 36.06 36.07 |

STEMI: ST Elevation Myocardial Infarction
PTCA: Percutaneous Transluminal Coronary Angioplasty
ER: Emergency Room
HEIS: Healthcare Emergency Information System
HDR: Italian Hospital Discharge Registry

**Table S3.** Link to indicators protocols for AMI pathways.

| N | Indicators | Link to indicator protocol |
|---|---|---|
| 1 | AMI: VOLUME OF HOSPITALIZATIONS | https://www.dep.lazio.it/prevale2021/documenti/protocolli/pro_122.pdf |
| 2 | STEMI: VOLUME OF HOSPITALIZATIONS | https://www.dep.lazio.it/prevale2021/documenti/protocolli/pro_98.pdf |
| 3 | STEMI: VOLUME OF HOSPITALIZATIONS WITH AT LEAST ONE PTCA | https://www.dep.lazio.it/prevale2021/documenti/protocolli/pro_206.pdf |
| 4 | STEMI: PROPORTION TREATED WITH PTCA WITHIN 90 MINUTES (INPATIENT FACILITY) | https://www.dep.lazio.it/prevale2021/documenti/protocolli/pro_610.pdf |
| 5 | STEMI: PROPORTION OF PTCA WITHIN 90' OF TOTAL TREATED WITH PTCA WITHIN 12H | https://www.dep.lazio.it/prevale2021/documenti/protocolli/pro_33.pdf |
| 6 | AMI: 30-DAY MORTALITY (INPATIENT FACILITY) | *Available upon request* |
| 7 | STEMI: 30-DAY MORTALITY (INPATIENT FACILITY) | *Available upon request* |
| 8 | AMI: 30-DAY MORTALITY | https://www.dep.lazio.it/prevale2021/documenti/protocolli/pro_1.pdf |
| 9 | STEMI: 30-DAY MORTALITY | https://www.dep.lazio.it/prevale2021/documenti/protocolli/pro_7.pdf |

**Table S4.** Link to indicators protocols for ischemic stroke pathways.

| N | Indicators | Link to indicator protocol |
|---|---|---|
| 1 | ISCHEMIC STROKE: VOLUME OF HOSPITALIZATIONS | https://www.dep.lazio.it/prevale2021/documenti/protocolli/pro_125.pdf |
| 2 | ISCHEMIC STROKE: NUMBER OF THROMBOLYSIS TREATMENTS AT THE FACILITY | *Available upon request* |
| 3 | ISCHEMIC STROKE: 30-DAY HOSPITAL READMISSIONS | https://www.dep.lazio.it/prevale2021/documenti/protocolli/pro_19.pdf |
| 4 | ISCHEMIC STROKE: IN-HOSPITAL MORTALITY | *Available upon request* |
| 5 | ISCHEMIC STROKE: 30-DAY MORTALITY AFTER FIRST ACCESS | https://www.dep.lazio.it/prevale2021/documenti/protocolli/pro_18.pdf |

**Figure S1: Number of hospitalizations of patients with STEMI by facility (2021).**

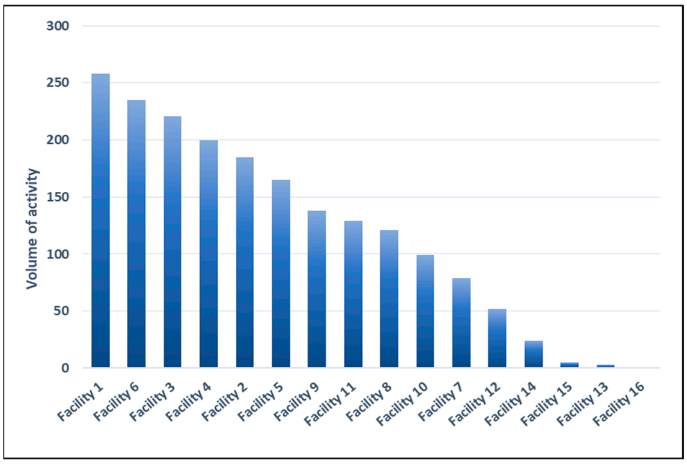


**Table S5:** Number of hospitalizations of patients with STEMI by facility (2021, 2020, 2019).

| Facility | Volume of Activity | | |
|---|---|---|---|
| | 2021 | 2020 | 2019 |
| **Lazio** | **3249** | **3219** | **3658** |
| Facility 1 | 258 | 299 | 410 |
| Facility 6 | 235 | 210 | 241 |
| Facility 3 | 221 | 204 | 270 |
| Facility 4 | 200 | 177 | 186 |
| Facility 2 | 185 | 150 | 193 |
| Facility 5 | 165 | 186 | 197 |
| Facility 9 | 138 | 132 | 182 |
| Facility 11 | 129 | 133 | 132 |
| Facility 8 | 121 | 126 | 126 |
| Facility 10 | 99 | 116 | 127 |
| Facility 7 | 79 | 83 | 101 |
| Facility 12 | 52 | 84 | 119 |
| Facility 14 | 24 | 9 | 19 |
| Facility 15 | 5 | 3 | 3 |
| Facility 13 | 3 | 11 | 8 |
| Facility 16 | 1 | 4 | 1 |

**Figure S2:** Proportion of patients with STEMI treated with PTCA within 90 min from access to ER of total treated with PTCA within 12h, by facility (2021).

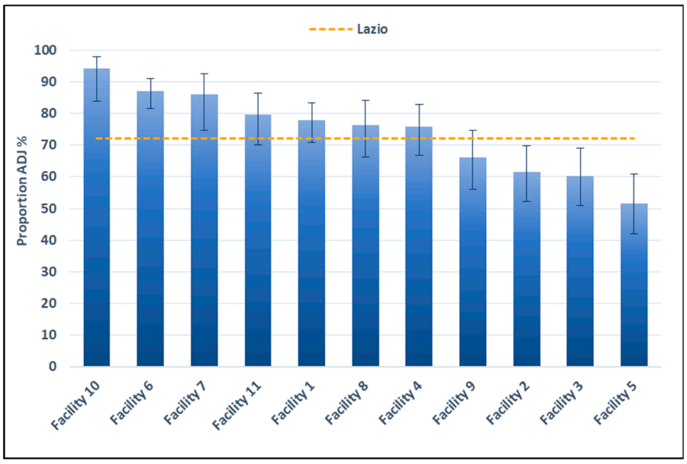


**Table S6:** Proportion of patients with STEMI treated with PTCA within 90 min from access to ER of total treated with PTCA within 12h, by facility (2021).

| Facility | N | Crude % | 95%CI | | Adj % | 95%CI | |
|---|---|---|---|---|---|---|---|
| **Lazio** | **1997** | **72.26** | **70.25** | **74.18** | _ | _ | _ |
| Facility 10 | 52 | 94.23 | 84.36 | 98.02 | 94.25 | 83.85 | 98.03 |
| Facility 6 | 195 | 86.67 | 81.18 | 90.74 | 86.97 | 81.53 | 90.96 |
| Facility 7 | 57 | 84.21 | 72.64 | 91.46 | 85.96 | 74.79 | 92.62 |
| Facility 11 | 93 | 79.57 | 70.28 | 86.51 | 79.58 | 70.17 | 86.57 |
| Facility 1 | 171 | 78.36 | 71.6 | 83.87 | 77.86 | 70.94 | 83.51 |
| Facility 8 | 88 | 77.27 | 67.49 | 84.78 | 76.45 | 66.35 | 84.23 |
| Facility 4 | 111 | 76.58 | 67.89 | 83.48 | 75.76 | 66.81 | 82.9 |
| Facility 9 | 99 | 66.67 | 56.91 | 75.18 | 66.05 | 56.12 | 74.74 |
| Facility 2 | 118 | 62.71 | 53.72 | 70.91 | 61.45 | 52.3 | 69.85 |
| Facility 3 | 113 | 60.18 | 50.96 | 68.73 | 60.32 | 50.95 | 68.98 |
| Facility 5 | 106 | 51.89 | 42.48 | 61.17 | 51.46 | 41.95 | 60.86 |
| Facility 12 | 27 | 55.56 | 37.31 | 72.41 | _ | _ | _ |
| Facility 14 | 1 | 0.00 | _ | _ | _ | _ | _ |
| Facility 15 | 1 | 0.00 | _ | _ | _ | _ | _ |
| Facility 13 | _ | _ | _ | _ | _ | _ | _ |
| Facility 16 | _ | _ | _ | _ | _ | _ | _ |

**Figure S3:** Mortality within 30 days after first admission to hospital for AMI, by facility (2021).

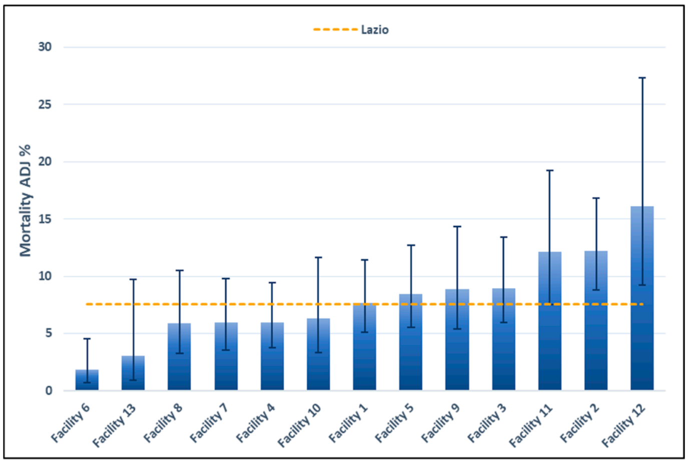


**Table S7:** Mortality within 30 days after first admission to hospital for AMI, by facility (2021).

| Facility | N | Crude % | 95%CI | | Adj % | 95%CI | |
|---|---|---|---|---|---|---|---|
| **Lazio** | **6302** | **7.55** | **6.93** | **8.23** | _ | _ | _ |
| Facility 6 | 303 | 1.65 | 0.71 | 3.80 | 1.84 | 0.74 | 4.52 |
| Facility 13 | 71 | 4.23 | 1.45 | 11.70 | 3.02 | 0.91 | 9.72 |
| Facility 8 | 262 | 4.58 | 2.64 | 7.83 | 5.92 | 3.29 | 10.52 |
| Facility 7 | 262 | 6.87 | 4.39 | 10.60 | 5.94 | 3.58 | 9.76 |
| Facility 4 | 358 | 5.87 | 3.87 | 8.80 | 5.96 | 3.74 | 9.43 |
| Facility 10 | 179 | 6.15 | 3.47 | 10.67 | 6.30 | 3.35 | 11.65 |
| Facility 1 | 421 | 6.41 | 4.44 | 9.17 | 7.68 | 5.12 | 11.44 |
| Facility 5 | 292 | 9.25 | 6.43 | 13.12 | 8.43 | 5.55 | 12.70 |
| Facility 9 | 232 | 7.76 | 4.96 | 11.93 | 8.86 | 5.41 | 14.31 |
| Facility 3 | 325 | 8.31 | 5.77 | 11.82 | 8.97 | 5.95 | 13.39 |
| Facility 11 | 183 | 10.93 | 7.19 | 16.28 | 12.12 | 7.50 | 19.24 |
| Facility 2 | 389 | 10.80 | 8.09 | 14.27 | 12.24 | 8.83 | 16.84 |
| Facility 12 | 102 | 14.71 | 9.12 | 22.85 | 16.12 | 9.21 | 27.32 |
| Facility 15 | 12 | 25.00 | 8.89 | 53.23 | _ | _ | _ |
| Facility 14 | 42 | 14.29 | 6.72 | 27.84 | _ | _ | _ |
| Facility 16 | 7 | 42.86 | 15.82 | 74.95 | _ | _ | _ |

**Figure S4:** In-hospital mortality within 30 days after first admission to hospital for STEMI, by facility (2021).

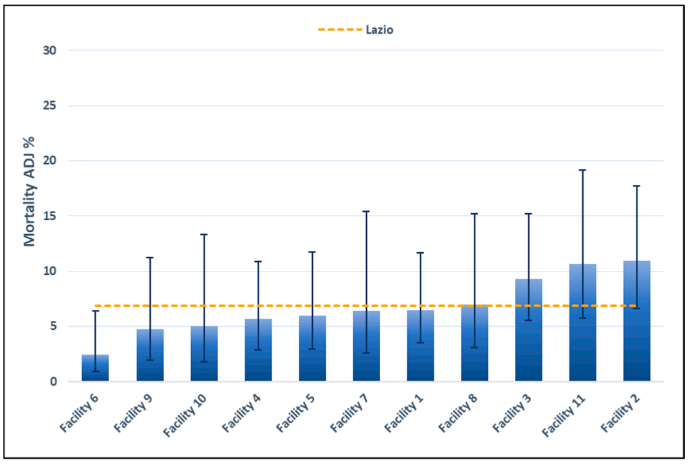


**Table S8:** In-hospital mortality within 30 days after first admission to hospital for STEMI, by facility (2021).

| Facility | N | Crude % | 95%CI | | Adj % | 95%CI | |
|---|---|---|---|---|---|---|---|
| **Lazio** | **2623** | **6.86** | **5.96** | **7.89** | _ | _ | _ |
| Facility 6 | 206 | 1.94 | 0.76 | 4.89 | 2.42 | 0.90 | 6.41 |
| Facility 9 | 122 | 4.10 | 1.76 | 9.24 | 4.73 | 1.94 | 11.22 |
| Facility 10 | 77 | 5.19 | 2.04 | 12.61 | 5.02 | 1.81 | 13.32 |
| Facility 4 | 164 | 6.10 | 3.35 | 10.86 | 5.68 | 2.90 | 10.90 |
| Facility 5 | 136 | 6.62 | 3.52 | 12.10 | 5.96 | 2.97 | 11.71 |
| Facility 7 | 71 | 7.04 | 3.05 | 15.45 | 6.41 | 2.56 | 15.42 |
| Facility 1 | 201 | 5.47 | 3.08 | 9.53 | 6.47 | 3.53 | 11.65 |
| Facility 8 | 110 | 5.45 | 2.52 | 11.39 | 6.94 | 3.06 | 15.17 |
| Facility 3 | 168 | 9.52 | 5.95 | 14.91 | 9.25 | 5.53 | 15.18 |
| Facility 11 | 109 | 11.01 | 6.41 | 18.26 | 10.69 | 5.78 | 19.16 |
| Facility 2 | 161 | 10.56 | 6.70 | 16.26 | 10.94 | 6.61 | 17.73 |
| Facility 12 | 39 | 23.08 | 12.65 | 38.34 | _ | _ | _ |
| Facility 15 | 3 | 33.33 | 6.15 | 79.23 | _ | _ | _ |
| Facility 14 | 6 | 0.00 | _ | _ | _ | _ | _ |
| Facility 13 | 2 | 0.00 | _ | _ | _ | _ | _ |
| Facility 16 | 1 | 0.00 | _ | _ | _ | _ | _ |

**Figure S5:** In-hospital mortality within 30 days after first admission to hospital for AMI, by facility (2021).

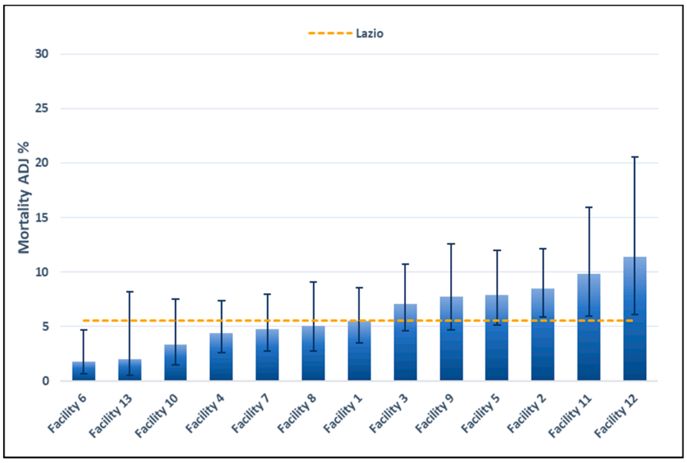


**Table S9:** In-hospital mortality within 30 days after first admission to hospital for AMI, by facility (2021).

| Facility | N | Crude % | 95%CI | | Adj % | 95%CI | |
|---|---|---|---|---|---|---|---|
| **Lazio** | **6302** | **5.52** | **4.98** | **6.11** | _ | _ | _ |
| Facility 6 | 303 | 1.32 | 0.51 | 3.34 | 1.77 | 0.66 | 4.70 |
| Facility 13 | 71 | 2.82 | 0.78 | 9.70 | 2.05 | 0.50 | 8.18 |
| Facility 10 | 179 | 3.35 | 1.55 | 7.12 | 3.36 | 1.48 | 7.49 |
| Facility 4 | 358 | 4.47 | 2.77 | 7.14 | 4.42 | 2.64 | 7.34 |
| Facility 7 | 262 | 5.73 | 3.50 | 9.23 | 4.74 | 2.78 | 7.99 |
| Facility 8 | 262 | 4.20 | 2.36 | 7.36 | 5.04 | 2.76 | 9.08 |
| Facility 1 | 421 | 4.75 | 3.10 | 7.22 | 5.48 | 3.49 | 8.55 |
| Facility 3 | 325 | 7.08 | 4.76 | 10.40 | 7.07 | 4.60 | 10.74 |
| Facility 9 | 232 | 7.33 | 4.62 | 11.42 | 7.74 | 4.70 | 12.58 |
| Facility 5 | 292 | 7.88 | 5.31 | 11.54 | 7.88 | 5.13 | 11.99 |
| Facility 2 | 389 | 8.23 | 5.89 | 11.38 | 8.48 | 5.88 | 12.12 |
| Facility 11 | 183 | 9.29 | 5.88 | 14.37 | 9.84 | 5.96 | 15.94 |
| Facility 12 | 102 | 10.78 | 6.13 | 18.29 | 11.36 | 6.08 | 20.53 |
| Facility 15 | 12 | 16.67 | 4.70 | 44.80 | _ | _ | _ |
| Facility 14 | 42 | 4.76 | 1.32 | 15.79 | _ | _ | _ |
| Facility 16 | 7 | 14.29 | 2.57 | 51.31 | _ | _ | _ |

**Figure S6:** Proportion of patients with STEMI treated with PTCA within 90 min from access to ER, by facility (2021). A: facility in volume class 0-10, B: facility in volume class 11-50, C: facility in volume class 51-100, D: facility in volume class 101-150, E: facility in volume class >=150.

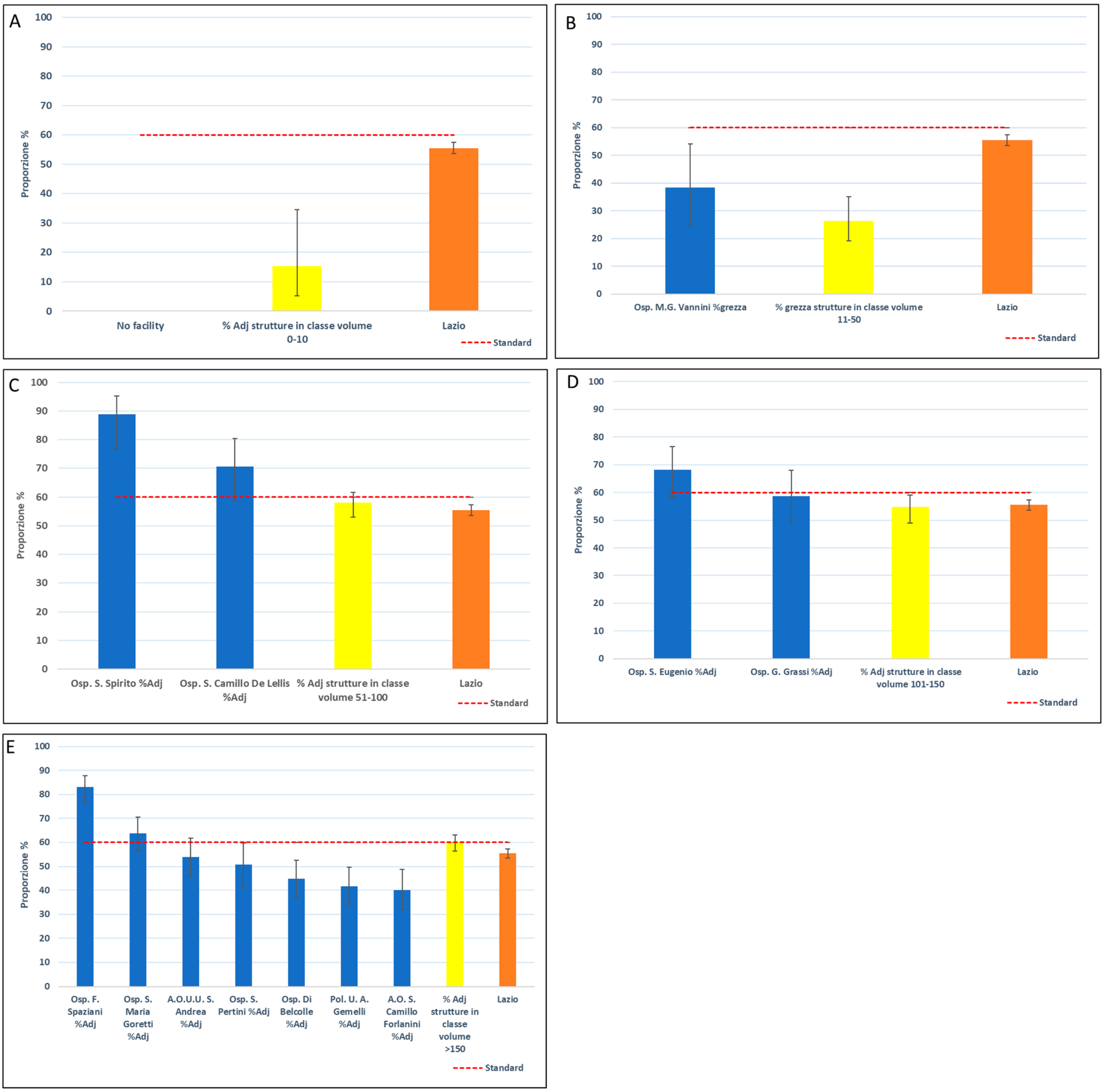

**Figure S7:** Mortality within 30 days after first admission to hospital for STEMI, by facility (2021). A: facility in volume class 0-10, B: facility in volume class 11-50, C: facility in volume class 51-100, D: facility in volume class 101-150, E: facility in volume class >=150.

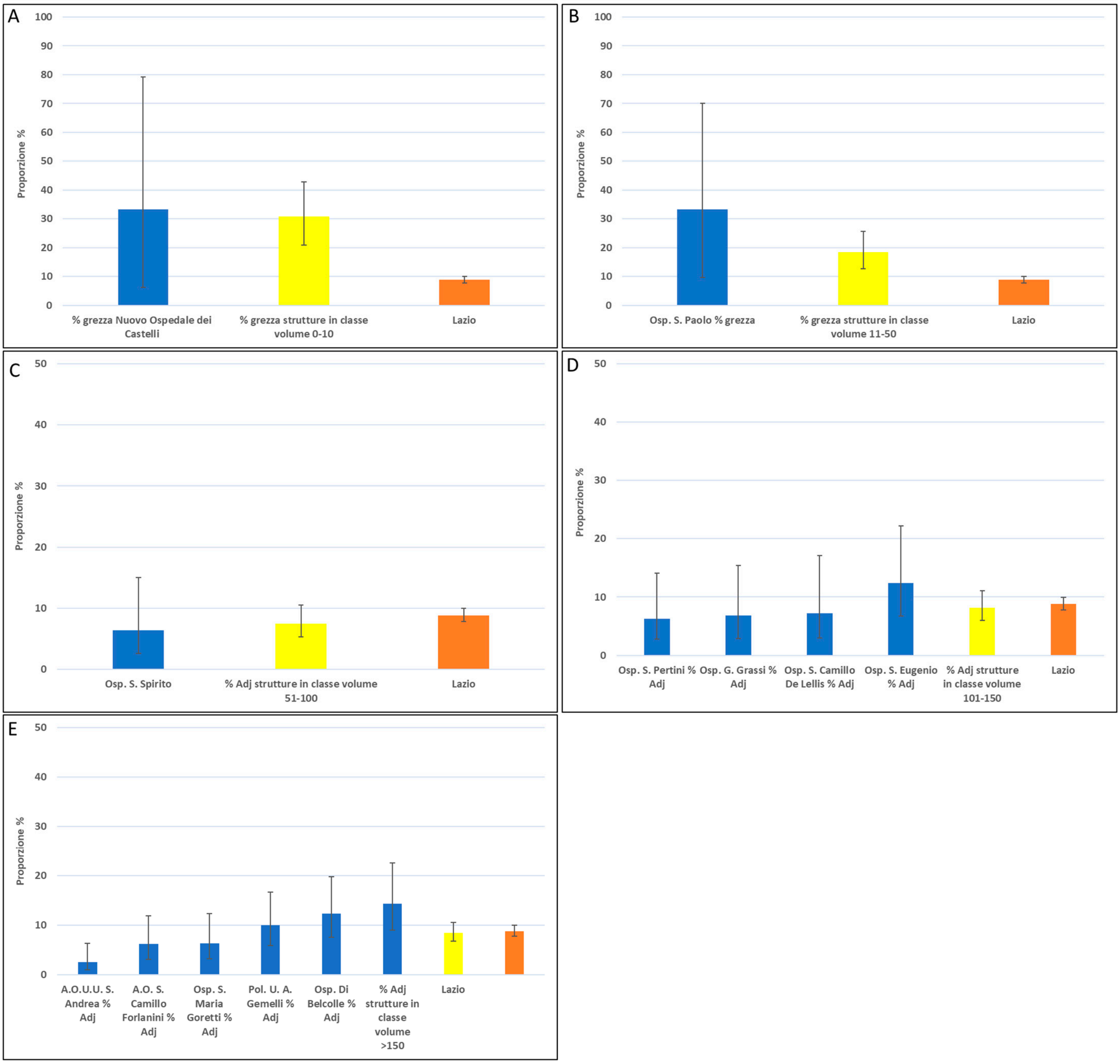

Supplement: Supplementary file 1 [file healthcare-11-01651-s001.zip › healthcare-2354467-supplementary.pdf]
